# Identification of an oral microbiota signature associated with an impaired orosensory perception of lipids in insulin-resistant patients

**DOI:** 10.1007/s00592-020-01567-9

**Published:** 2020-07-16

**Authors:** Philippe Besnard, Jeffrey E. Christensen, Arnaud Bernard, Isabelle Simoneau-Robin, Xavier Collet, Bruno Verges, Rémy Burcelin

**Affiliations:** 1 UMR 1231 Lipides/Nutrition/Cancer INSERM/Univ Bourgogne-Franche Comté/AgroSup Dijon, 21000 Dijon, France; 2 UMR 1048 INSERM/Univ Toulouse III Paul Sabatier, 31000 Toulouse, France; 3 Physiologie de La Nutrition, AgroSup Dijon, 26 Bd Dr Petitjean, 21000 Dijon, France

**Keywords:** Microbiota, Circumvallate papillae, Taste sensitivity, Lipids, Type 2 diabetes

## Abstract

**Aims:**

Type 2 diabetes leads to multiple sensory dysfunctions affecting notably the gustatory sensitivity. Although this sensory defect, by impacting food choices, might lead to unhealthy eating behavior, underlying mechanisms remains poorly studied. We have recently reported that the composition of microbiota in contact with circumvallate gustatory papillae might affect the orosensory perception of lipids in lean and normoglycemic obese subjects. This finding has prompted us to explore whether such a phenomenon also occurs in diabetic obese patients.

**Methods:**

The composition of microbiota surrounding the circumvallate papillae was analyzed in combination with the linoleic acid perception thresholds in male insulin-resistant patients and weight-matched healthy controls*.* Two complementary comparisons were performed: (1) controls *vs* diabetic and (2) diabetic low-lipid tasters versus diabetic high-lipid tasters.

**Results:**

Despite subtle modifications in the oral microbiota composition, comparison of orosensory lipid perception in controls and diabetic subjects did not lead to discriminating data due to the large inter-individual variability of linoleic acid perception thresholds. In contrast, specific bacterial signatures were found by comparing diabetic low- and high-lipid tasters leading to differential molecular pathways. Surprisingly, a lower fatty taste perception was mainly found in patients treated with metformin and/or statins, suggesting a possible side effect of these antidiabetic and/or hypolipidemic drugs on taste acuity.

**Conclusions:**

Collectively, these data show that the diabetic patients with defective fatty taste detection are characterized by a specific microbiota metabolism at the circumvallate papillae levels, this occurrence seeming amplified by drugs commonly used to counteract the damaging metabolic effects of T2D.

Trial registration for original previous studies: ClinicalTrials.gov #NCT02028975

## Introduction

The prevalence of type 2 diabetes (T2D) has increased dramatically in the past decades becoming a major health issue worldwide. This obesity-related disease is associated with metabolic disorders at the origin of serious long-term health problems (heart disease, stroke, hypertension, eye and kidney damages, neuro-degeneration), mainly due to vascular and nerve impairments. Global sensory alterations are also commonly found in T2D patients, deeply impacting their quality of life. Lower tactile sensations [1], hearing deficit [2], visual [3] and olfactory [4] impairments are prevalent in most subjects with long-term uncontrolled glycemic levels [5]. Taste alterations have also been reported in T2D patients. Higher oral threshold response (i.e., lower taste sensitivity) to sweet, salty and sour stimuli were found in T2D patients as compared to BMI-matched healthy controls, while their orosensory detection of bitter remained unchanged [6, 7]. Since sweet and salt tastes are strong contributors of hedonic value of foods, this gustatory blunting might lead to eating behavior changes in order to reach the expected food reward. Consistent with this assumption, cravings for sugar-rich foods were more common in patients with T2DM than in age-, sex- and BMI-matched controls, this phenomenon being tightly related to a poor glycemic control [8].

Although lipids also render foods highly palatable and the existence of a fatty taste is supported by a growing number of studies, it is not yet known whether T2D also can affect the orosensory fat perception**.** Likewise, the molecular mechanism by which T2D affects the taste sensitivity remains poorly known. Nevertheless, several lines of evidence suggest an implication of oral microbiome. Firstly, systemic inflammatory diseases, including diabetes, are associated with an oral dysbiosis [9]. Secondly, an association between the composition of oral microbiota surrounding the gustatory circumvallate papillae (CVP) and orosensory lipid perception is found in normoglycemic obese patients [10]. Thirdly, gut [11] and oral [12] dysbiosis found in T2D induces a resistance to GLP-1 [13], an incretin known to be a key regulator of both sweet and fatty taste sensitivity. Indeed, the GLP-1 analogue Liraglutide increases the sweet taste sensitivity and decreases the preference for fatty foods in patients with poorly controlled T2D [14]. Moreover, disruption of the GLP-1 gene is associated with a reduction of orosensory sensitivity to lipids in the mouse [15]. Collectively, these data raise the question of impact of T2D on the fatty taste sensitivity and oral microbial environment surrounding gustatory papillae. To explore this issue, the oral perception threshold of a fatty acid widely found in foods (i.e., linoleic acid—LA) and microbiota composition in the direct vicinity of gustatory papillae having the higher density of lingual taste buds (i.e., CVP) were compared in normoglycemic weight-matched obese controls (C) and T2D (D) patients.

## Materials and methods

### Subjects

**Fig. 1:**
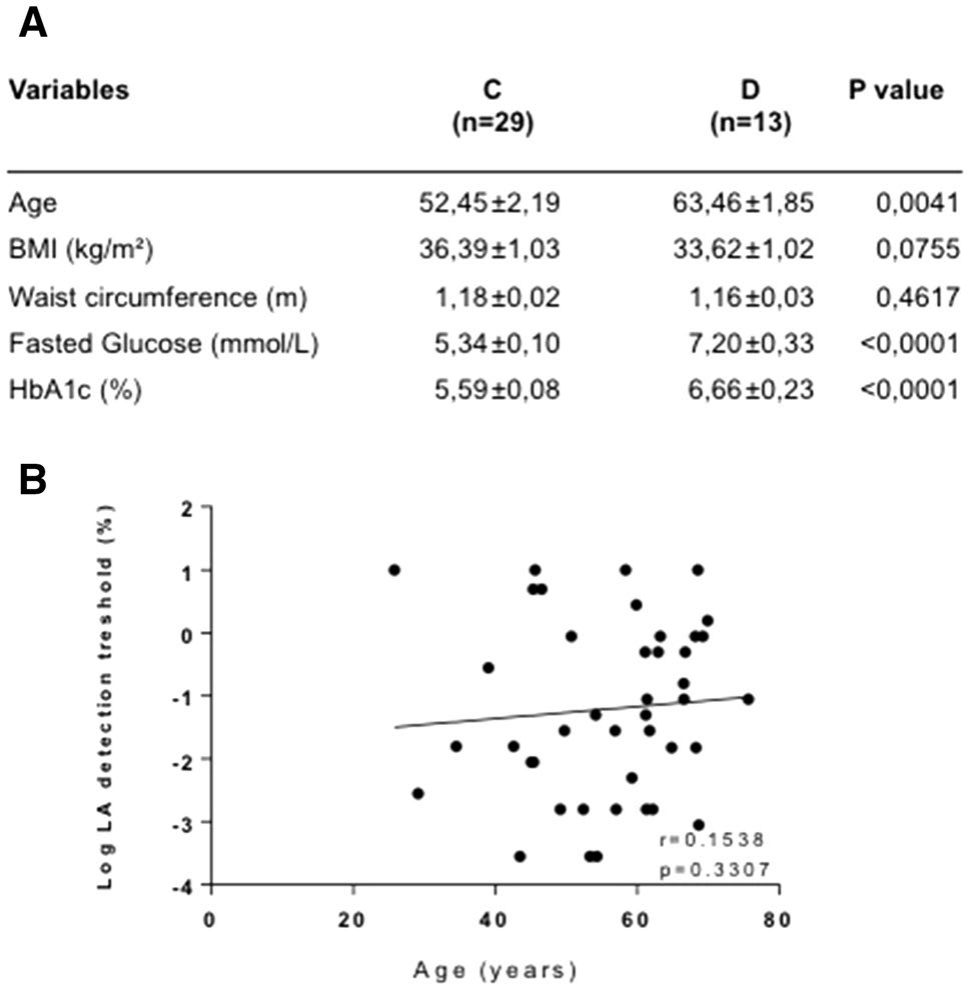
Phenotyping of controls (C) and type 2 diabetic (D) patients. **a** Comparison of main clinical variables. **b** Investigation of aging effect on the oral linoleic acid (LA) perception thresholds

Forty-two male subjects, 26–74 years of age, participating to the HumanFATaste program were included in this study. All subjects received detailed information about the study and gave written consent (ANSM n° B90706-40). HumanFATaste was approved by the local ethics committee (Comité de Protection des Personnes—CPP Est1, n°2009/18) and registered at ClinicalTrials.gov (#NCT02028975). T2D patients (D, *n* = 13) displayed a BMI ≥ 30 kg/m^2^, a fasted glycemia > 6.10 mmol.L^−1^ and a glycated HbA1c > 6.0% were included in this study (Fig. 1a). Weight-matched normoglycemic controls (C, *n* = 29) were selected according to the following inclusion criteria: fasted glycemia < 6.10 mmol.L^−1^, glycated hemoglobin A1c < 6.0%, no hypoglycemic drugs intake or surgical treatment of obesity. Smokers or former smokers (for less than 3 months) were excluded as well as subjects with severe digestive pathologies, pancreatic, renal, hepatic failures, type 1 diabetes, patients treated with inhibitors of proton pumps, insulin, GLP-1 analogs or DPP-IV inhibitors (Fig. 1b). The diabetic patients included in the study followed the nutritional recommendations from the Société Francophone du Diabète (SFD) advising a diet including 50–55% carbohydrates with low glycemic index, 35–40% lipids with less than 10% of saturated fat and 25–30% of monounsaturated fat and polyunsaturated fat, 25 g/day of fiber and 2 to 3 fruits per day (https://www.sfdiabete.org/recommandations/referentiels).

### Oral lipid perception

Determination of orosensory perception threshold of lipids was performed using the three-alternative forced-choice test [16] with linoleic acid (LA) as a lipid model [17]. To limit ingestive and post-ingestive interferences on oral lipid perception, tests were performed in the morning in fasted subjects. The protocol used is fully detailed elsewhere [17]. In brief, to minimize textural cues between control and experimental solutions (from 0.00028 to 5% LA wt/wt, spaced by 0.25 log units), 5% acacia gum and 5% mineral oil (wt/wt) were added in mineral water. Subjects had to identify the LA solution among 3 samples. Each sample (5 ml) was kept in the mouth for 7 s then spit out, before tasting the next sample 20 s later. The use of alimentary dyes demonstrated that this protocol allows the contact of the solutions with whole the dorsal tongue including the circumvallate papillae (CVP). Sets were offered in an ascending concentration of LA with a break of 60–120 s between 2 sets, allowing a mouth rinsing with water. The procedure was stopped when a LA sample was correctly identified 3 times, consecutively. This concentration constitutes the LA perception threshold of the subject. Testing was conducted under red lighting and with participants wearing a nose clip to limit visual and olfactory inputs, respectively. Despite the wide age range (26–74 years), aging was not found to affect significantly the oral LA thresholds (Fig. 1b).

### Oral microbiota samples and analysis

Swab samples were taken directly from the V-shaped row of the CVP at the posterior part of the tongue dorsum to collect associated microbiota. These gustatory papillae were preferred to fungiforms and foliates papillae because the CVP house the most of the taste buds are found lingual epithelium. Moreover, in contrast to other gustatory papillae, the CVP display a dome-shaped structure with a circular cleft favorable to development of specific multispecies bacterial communities. Consistent with this assumption, a recent analysis of microbiota distribution across the human dorsal tongue highlighted a complex spatial organization with multiple microbial consortia [18].

### Oral microbiota analysis

Total bacterial DNA was extracted as previously described [19]. The bacterial population present in the samples has been determined using next generation high throughput sequencing of variable regions of the 16S rRNA bacterial gene, with a specific protocol established, as described (Vaiomer SAS www.vaiomer.com). The metagenomics workflow was used to classify organisms from a metagenomic sample by amplifying specific regions in the 16S ribosomal RNA gene and was exclusive to bacteria. V3-V4 hypervariable regions of the 16S rDNA gene were amplified from the DNA extracts during a first PCR step using universal 16S primers V2, as described [19]. The targeted metagenomic sequences from microbiota were analyzed using an established bioinformatics pipeline [19].

### Statistical analysis

For the peri-papillae microbiota, the output matrix containing the relative abundance of OTUs per sample was processed with the LEfSe (linear discriminant analysis effect size) algorithm 30 using an alpha cutoff of 0.05 and an effect size cutoff of 2.0. Statistical analyses (nonparametric Mann–Whitney’s tests and nonparametric Kruskal–Wallis tests followed by Dunn’s multiple comparison tests and Spearman’s correlations) were conducted using the software PRISM v6.05 and the software environment R version 3.3.1.

## Results

### Controls and diabetic patients display a large inter-individual variability in both LA perception and oral microbiota

**Fig. 2:**
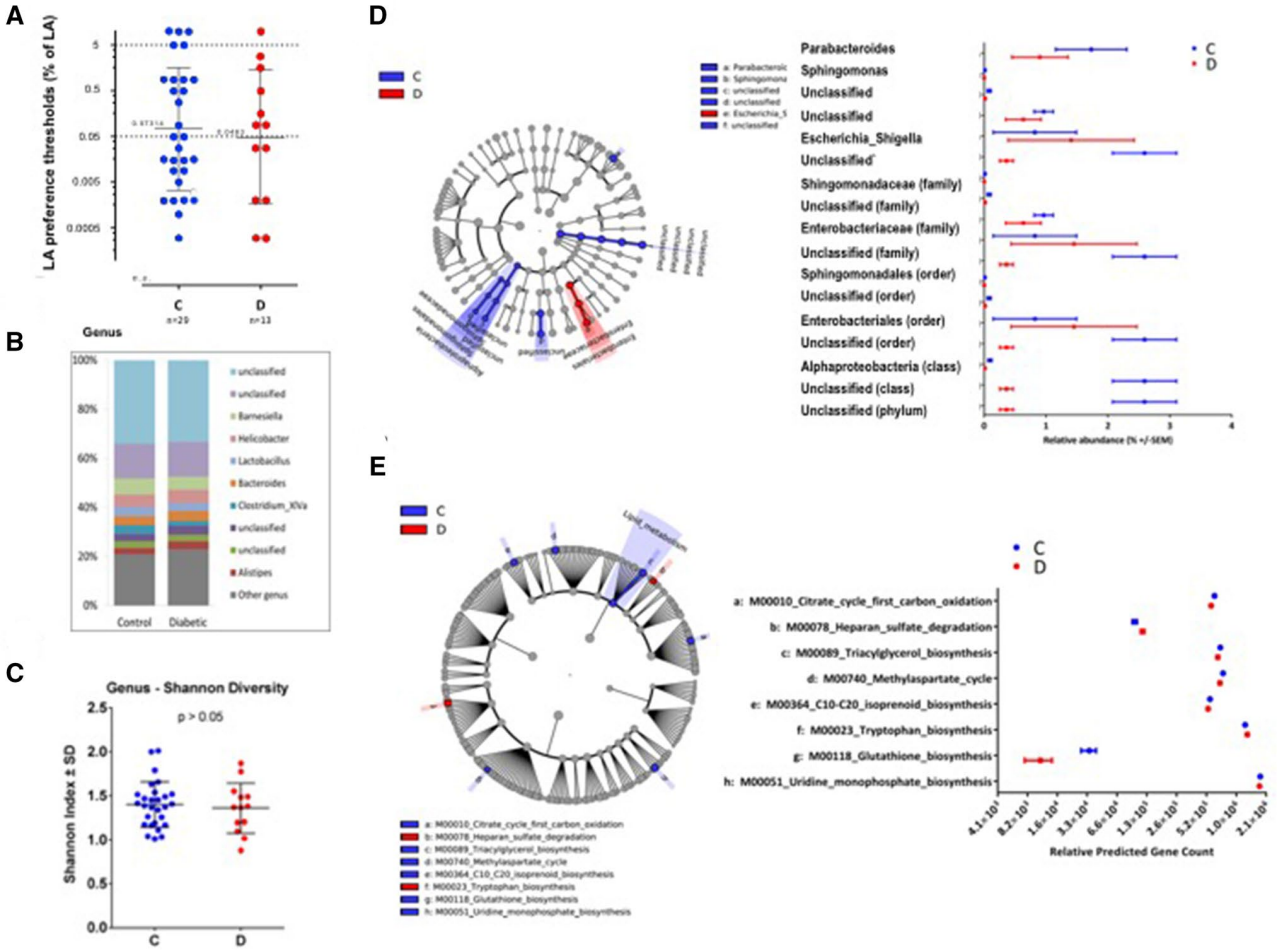
Orosensory and microbiome analysis of obese control (C) and diabetic patients (D). **a** Linoleic acid (LA) perception thresholds. **b** Distribution of the most abundant resolved taxa at the genus level. **c** Microbiota alpha diversity. **d** Cladogram analysis of 16S sequences from circumvallate papillae and histogram showing the taxonomic groups (*P* < 0.05; LDA score 2.0). **e** Cladogram derived from KEGG pathway contributions of predicted metagenomic data. The relative predicted gene count histogram shows the KEGG pathway modules (*P* < 0.05; LDA score 2.0)

In a first attempt to explore the fatty taste sensitivity and identify CVP microbiota differences between groups, we determined the LA perception thresholds and analyzed the taxonomic identity of the individuals. Consistent with previous studies [16, 17], we observed a large inter-individual variability in LA perception thresholds that led to a lack of significant difference between obese C and D patients (Fig. 2a). No significant difference in composition (Fig. 2b) and alpha diversity (Shannon index—Fig. 2c) at any taxonomic level of microbiota surrounding the CVP allowed discriminating C from D groups. In a second attempt, we performed a more thorough analysis using LEfSe and identified six genus level differential features between the C and D (Fig. 2d). A high proportion of this difference was due to an increased frequency of *Parabacteroides* in the C group and an increased frequency of *Escherichia_Shigella* in the D group, suggestive of a first signature, although subtle, of the diabetic status. Additionally, a large difference was observed between the groups for a yet unclassified genus, which was elevated in the C (2.6% vs. 0.4%). However, the majority of this unclassified genus was comprised of counts for OTU_00018, identified as unclassified (phylum) due to a low confidence threshold of 80% for the phylum *Proteobacteria* (RDP Classifier). To infer the putative metabolomic pathways differentially expressed between groups, we performed a metagenomics prediction using PICRUSt, and the relative abundance of KEGG modules for metabolic pathway and structural complex features were determined for C versus D. A LEfSe analysis identified 8 KEGG modules, highlighting differential metabolic pathway features between the C and D groups, with a high proportion of this difference due to higher counts for glutathione biosynthesis, carbohydrate and lipid metabolism in the C group (Fig. 2e), further supportive of a specific signature of the diabetic group. It is, however, noteworthy that the metabolic signature concerns, at least in part, the lipid metabolism. However, this first analysis does not preclude differences between taster and non-taster patients inferring the aggravation of the diabetic phenotype.

### CVP microbiota composition in diabetic low- and high-LA tasters

**Fig. 3:**
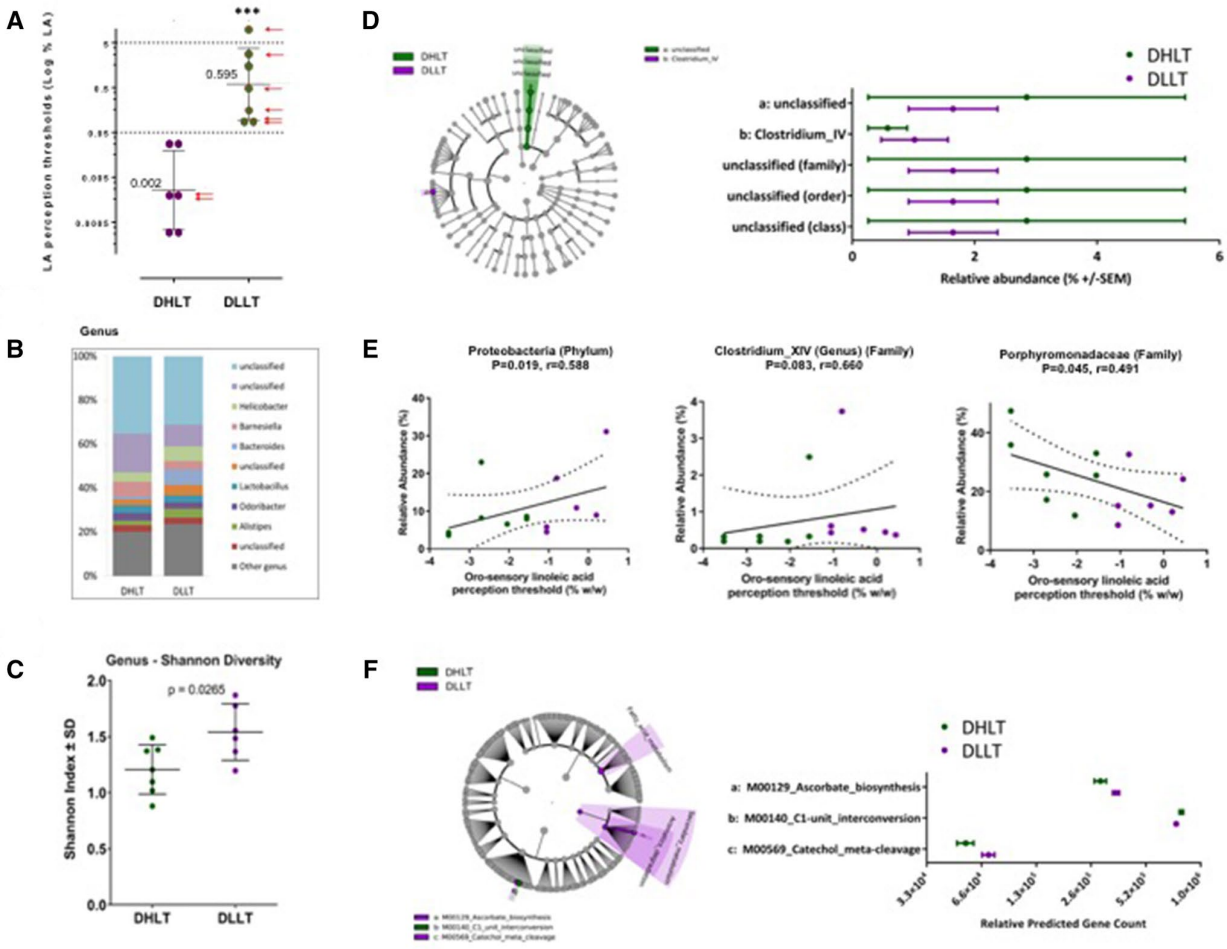
Orosensory and microbiome analysis of diabetic high-lipid tasters (DHLT) and diabetic low-lipid tasters (DLLT). Panel **a** Orosensory perception threshold of linoleic acid (LA). **b** Distribution of the most abundant resolved taxa at the genus level. **c** Microbiota alpha diversity. **d** Cladogram analysis of 16S sequences from circumvallate papillae and histogram showing the taxonomic groups (*P* < 0.05; LDA score 2.0). **e** Linear regression analyses between the frequency of identified bacterial taxa and perception threshold of LA. **f** Cladogram derived from KEGG pathway contributions of predicted metagenomic data. The relative predicted gene count histogram shows the KEGG pathway modules (*P* < 0.05; LDA score 2.0). Means ± SEM, *P* < 0.001. *M* metformin, *S* statins (color figure online)

To further explore the putative links between CVP microbiota composition and orosensory perception thresholds of lipids, we therefore postulated that D subjects displaying a threshold value ≤ 0.05% LA were high-LA tasters (DHLT), others being low-LA tasters (DLLT—Fig. 3a). This new analysis led to discriminant data. The difference in the LA perception thresholds found between DLLT and DHLT groups (Fig. 3a) was also associated with changes in the CVP microbiota composition (Fig. 3b). Few significant differences were observed for the 10 highest relative abundance taxa at the genus level (Fig. 3b). DLLT group was notably characterized by an increased abundance of *Bacteroidaceae* (*Bacteroides* genus) and *Helicobacter*, and a reduced presence of *Barnesiella*. There was a statistically significant difference in alpha diversity between DLLT and DHLT groups, but only at the genus level (*p* = 0.0265), with a higher diversity in the DLLT group (Fig. 3c). The LEfSe analysis comparison of DLLT and DHLT groups identified two genus level differential features, with a high proportion of this difference due to unclassified bacteria of the phylum *Bacteroidetes* (Fig. 3d). A significant positive correlation with LA detection threshold (i.e*.*, a lower sensitivity) was observed for the phylum Proteobacteria and genus *Clostridium_XIV* while a negative correlation (i.e., a higher sensitivity) was found for the family *Porphyromodaceae* (Fig. 3e), suggesting that the composition of the CVP microbiota might affect the fatty taste sensitivity. Predicted metagenomics was performed using PICRUSt and the relative abundance of KEGG modules for metabolic pathways and structural complex features were determined for DLLT versus DHLT. A LEfSe analysis identified 3 KEGG module levels with differential metabolic pathway features between the DLLT and DHLT groups, with a high proportion of this difference due to 2 modules related to counts for ascorbate and catecholamine pathways (Fig. 3f). Unexpectedly, LA thresholds analysis taking into account treatments of patients suggested that fatty taste sensitivity might be decreased following administration of antidiabetic and/or hypolipidemic drugs, metformin (*M*) and statins (*S*) (Fig. 3a, red arrows).

## Discussion

Despite a relative limited number of T2D patients, the present study brings several original information. Firstly, a similar inter-individual heterogeneity of LA perception threshold was found in D patients and in weight-matched controls suggesting that insulin resistance, as such, does not play a major role in the modulation of fatty taste sensitivity. Therefore, T2D might differentially affect taste modalities since the detection of sweet, salty and sour stimuli are found to be decreased in D patients, whereas bitter perception remains unchanged [7], as reported herein for lipids. The origin of this differential impact remains to be elucidated. In contrast, analysis of microbiota in the direct vicinity of CVP reveals some changes discriminating D from C subjects. Notably at the metabolic level, the diabetic status is characterized by a decrease of glutathione (GSH) biosynthesis. The fact that a GSH deficiency was recently reported in T2D patients [20] and the GSH synthesis was modulated by the gut microbiota through its action on the host amino acid metabolism [21] is in agreement with our observation. Although GSH is a major cellular antioxidant preventing tissues against the reactive oxygen species damages, the putative GSH deficit at the CVP level does not impact the fatty taste sensitivity of D patients.

Secondly in T2D patients, the existence of variations in the bacterial composition surrounding the CVP allows to distinguish low- from high-lipid tasters. DLLT subjects are especially characterized by a greater bacterial diversity and a proinflammatory profile with a high *bacteroides/lactobacillus* ratio as compared to DHLT. Such a bacterial environment might interfere with the oral fat perception since obesity-induced inflammation reduces the taste bud density and turnover in the mouse [22], an event known to specifically reduce the fatty taste sensitivity in human [23]. Moreover, differences in bacterial metabolism involving catecholamine and ascorbate pathways were found to be higher in DLLT patients than in DHLT patients. Whether this metabolic signature affects the oral perception of lipids is unknown. However, the fact that (1) *Clostridia* play a critical role in the generation of free catecholamines [24], (2) a positive correlation (i.e*.*, low LA sensitivity) between *Clostridium_XIV* and LA perception threshold found in D patients (Fig. 3e), and (3) bitter and sour taste perception thresholds are reduced by norepinephrine in humans [25] raises this possibility. Further studies are required to explore this hypothesis.

Thirdly, it was found that the most of DLLT patients were treated with metformin and/or statins (Fig. 3a, red arrows). This intriguing observation is consistent with previous studies reporting that these drugs elicited taste-related adverse effects, i.e., dysgeusia [26, 27], and impacted the gut microbiota ecology [28]. Whether these drugs can also affect the oral microbiota composition is not known. Despite the major limitation due to a very small number of patients, this unexpected observation has the merit of questioning the potential impact on the taste perception of treatments commonly used in insulin-resistant patients and of proposing a mechanistic track.

In conclusion, this exploratory study consolidates our previous investigation showing the existence of relationships between microbiota surrounding CVP and fatty taste sensitivity [10]. It also provides the original information that T2D patients unable to properly detect low-lipid stimuli (DLLT) displayed a specific microbiota signature at the CVP levels, this occurrence seeming amplified by drugs commonly used to counteract the damaging metabolic effects of T2D. By modifying the oral perception of foods, these sensory defects might make difficult the compliance with healthy nutritional recommendations. A better understanding of molecular mechanisms connecting T2D and sensory disorders might lead to new therapeutic strategies improving the quality of life of patients.

## Abbreviations

C: Controls
CVP: Circumvallate papillae
D: Diabetic
DHLT: Diabetic high-linoleic acid tasters
DLLT: Diabetic low-linoleic acid tasters
GSH: Glutathione
LA: Linoleic acid
T2D: Type 2 diabetes
